# Video-Based Person Re-Identification by an End-To-End Learning Architecture with Hybrid Deep Appearance-Temporal Feature

**DOI:** 10.3390/s18113669

**Published:** 2018-10-29

**Authors:** Rui Sun, Qiheng Huang, Miaomiao Xia, Jun Zhang

**Affiliations:** School of Computer Science and Information Engineering, Hefei University of Technology, Feicui Road 420, Hefei 230000, China; sunrui@hfut.edu.cn (R.S.); 18225514947@163.com (M.X.); zhangjun@hfut.edu.cn (J.Z.)

**Keywords:** person re-identification, end-to-end architecture, appearance-temporal features, Siamese network, pivotal frames

## Abstract

Video-based person re-identification is an important task with the challenges of lighting variation, low-resolution images, background clutter, occlusion, and human appearance similarity in the multi-camera visual sensor networks. In this paper, we propose a video-based person re-identification method called the end-to-end learning architecture with hybrid deep appearance-temporal feature. It can learn the appearance features of pivotal frames, the temporal features, and the independent distance metric of different features. This architecture consists of two-stream deep feature structure and two Siamese networks. For the first-stream structure, we propose the Two-branch Appearance Feature (TAF) sub-structure to obtain the appearance information of persons, and used one of the two Siamese networks to learn the similarity of appearance features of a pairwise person. To utilize the temporal information, we designed the second-stream structure that consisting of the Optical flow Temporal Feature (OTF) sub-structure and another Siamese network, to learn the person’s temporal features and the distances of pairwise features. In addition, we select the pivotal frames of video as inputs to the Inception-V3 network on the Two-branch Appearance Feature sub-structure, and employ the salience-learning fusion layer to fuse the learned global and local appearance features. Extensive experimental results on the PRID2011, iLIDS-VID, and Motion Analysis and Re-identification Set (MARS) datasets showed that the respective proposed architectures reached 79%, 59% and 72% at Rank-1 and had advantages over state-of-the-art algorithms. Meanwhile, it also improved the feature representation ability of persons.

## 1. Introduction

Person re-identification (person Re-ID) aims at matching a target person across non-overlapping cameras at different times or different locations. It not only has important significance in video surveillance systems and the public security field, but is also a crucial challenge in the field of multi-camera visual sensor networks [1]. In real world situations, because multi-camera visual sensor networks capture the video clip of the target person, research on video-based person re-identification is necessary and inevitable for public safety. Video-based person re-identification is the task of utilizing a sequence of images/tracklets to match the person. At present, an increasing number of exiting research works [2,3,4,5] focus on video-based person re-identification.

More specifically, the process of video-based person Re-ID is to give a probe video and search the same person as the probe video in a large gallery of videos. As the probe video and gallery videos are taken from different cameras, they may suffer from inherent challenges such as lighting variations, camera viewpoint changes, background clutter or occlusions, and the person’s appearance similarity during person matching. In general, video-based person Re-ID is beneficial to improve the results of person Re-ID under the complex and difficult conditions described above. The reason for this fact is that video-based person Re-ID has the following advantages over still-image-based person Re-ID. Firstly, videos contain more information than a single still image contains. Given the availability of video clips, we can obtain temporal information related to a person’s motion. If the person suffers from problems including occlusion, background clutter, and appearance similarity, the person’s appearance information, based on a single still-image, is incomplete or missing. However, the use of potential temporal information based on image sequence can effectively alleviate the lack of motion information. What is more, videos provide a large number of the same person’s samples, so we can obtain more abundant appearance information to against camera viewpoint changes.

**Figure 1:**
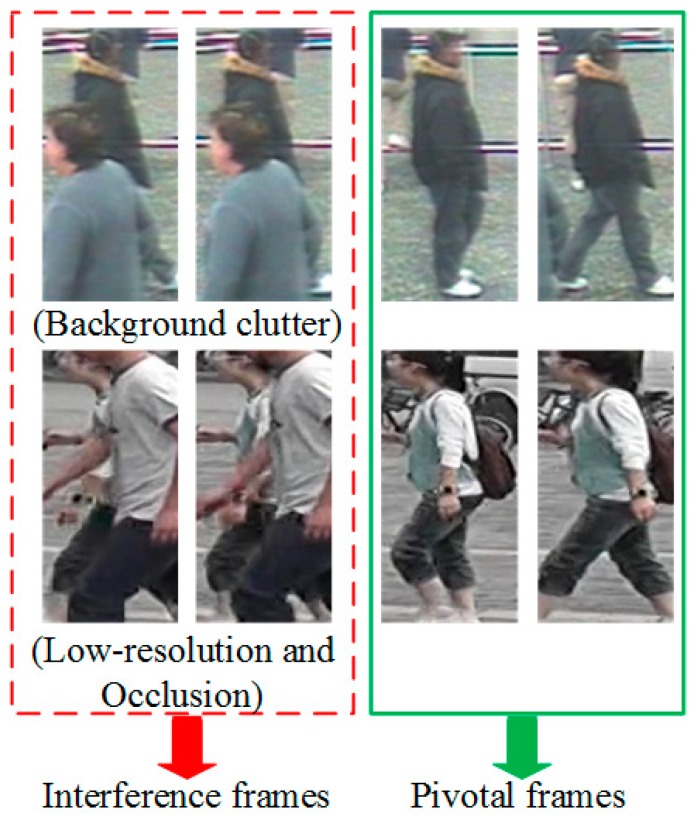
An illustration of the interference frames and pivotal frames definition. The green box indicates the pivotal frame (“good” frame).The red dotted box indicates the interference frame with low-resolution image, occlusion, and background clutter.

On the other hand, the use of video also brings several challenges for identifying the person. Firstly, some low-resolution image frames may appear in the captured video clips, which lead to inaccurate appearance information. Secondly, when the target pedestrian is obstructed or interfered with by objects or different persons in a video fragment, it becomes difficult to obtain the person’s appearance information in the current image sequence. Lastly, although the temporal information in the video is an important clue to identify pedestrians, the movement of different persons may also be similar, which means that purely using temporal information will cause misunderstanding. As shown in Figure 1, in this work, we define the appearance of ambiguity image frames and occluded image frames as interference frames in the video, and others image frames (“good” frames) that contain the full clear persons in the video as pivotal frames. Therefore, the following issues in video-based person Re-ID should be considered. (1) How to establish a stable pedestrian appearance representation model, that enables elimination of the effects of interference frames on individuals’ representation in videos? (2) How to effectively harness two types of complementary information including appearance features and temporal features in the video to compare the degree of similarity between different persons, so that the role of pivotal frames is fully realized?

To address the first problem, for one thing, previous works have adopted new features [6], appearance feature models, and semantic attribute features [7,8], which extract robust and discriminative information to represent a person. However, we can observe that not all images are informative in a given video, and severe interference frames cause previous methods to obtain erroneous information. For another thing, the current common research idea [5,9] is to adopt a combination of convolutional neural network (CNN) and recurrent neural network (RNN) to extract the space-time features of each image frame, and aggregate them into a single feature vector by the pooling operation. Although these methods have achieved good results, the interference frames in the video will influence the final feature information. Simultaneously, such methods do not make full use of the person’s appearance information. To sum up, in this work, we propose a Two-branch Appearance Feature (TAF) sub-structure which consists of the walking cycle analysis model [2], the two-branch Inception-V3 network, and the fusion layer, to select pivotal frames (“good” frames) and discard interference frames, then learn the global and local discriminative appearance feature information.

To deal with the second problem, the current work [10] mainly focuses on the integration of two types of features before learning the distance between persons. Appearance features and temporal features are different modal information. We believe that information maybe lost due to information inequality when these two types of features are combined. In this paper, inspired by a previous literature [11], instead of merging the temporal features and the appearance features of pivotal frames, we learn the independent distances of the two types of features separately. Hence, we designed a hybrid end-to-end deep learning architecture for further learning the feature representation and the independent distance metric. The hybrid end-to-end architecture consists of a two-stream appearance-temporal deep feature structure and two Siamese networks. The integrated architecture separately obtains the person’s appearance features and temporal features through the hybrid feature structure, whilst using the two Siamese networks to learn the independent distances of the two types of features.

In summary, the main contributions of this paper are three-fold as follows.

(1) We propose a Two-branch Appearance Feature (TAF) sub-structure consisting of the walking cycle model, the two-branch Inception-V3 network, and the saliency learning fusion layer, which is used to learn the global and local appearance features of persons. This sub-structure is useful for discarding interference frames with occlusion and background clutter in the video, and selecting informative pivotal frames. The features of these pivotal frames can promote the representation learning ability of two-branch Inception-V3 network. Simultaneously, the fusion layer can improve the fusion effect and the learning result of local information.
(2) We design a two-stream hybrid end-to-end deep learning architecture that combines feature learning and metric learning, which uses a hybrid deep feature structure and two Siamese networks to obtain a person’s features and separately achieve the independent distance metric of appearance features and temporal features. Note that it can obtain better appearance information and temporal information by having two independent feature sub-structures.
(3) We evaluate our proposed architecture on three public video datasets, including PRID-2011 dataset [12], iLIDS-VID dataset [13], and MARS (Motion Analysis and Re-identification Set) dataset [14]. Extensive comparative experiments show that our proposed video-based person Re-ID architecture achieves comparable results to the existing state-of-the-art methods.

The remainder of this paper is organized as follows. Section 2 reviews the work related to person Re-ID. Section 3 gives a complete explanation of the architecture proposed in this paper and a detailed introduction to each part of the architecture. Section 4 conducts an experimental evaluation of the performance of the proposed algorithm on public datasets. Finally, Section 5 summarizes the work of this paper.

## 2. Related Work

Person re-identification has attracted the attention of many researchers in recent years. With the development of person re-identification works, we believe that the study of person re-identification can be roughly divided into three groups: image-based person re-identification [15,16,17,18,19,20,21], video-based person re-identification [2,3,4,5,9,22], and image to video person re-identification [23]. Typically, most existing person Re-ID algorithms focus on three key steps: feature extraction [15,16,17,18,19], distance measure [20,21], and end-to-end learning methods [11,24,25,26,27]. To obtain reliable feature representations, the features adopted in the existing person Re-ID work can be divided into hand-designed features [15,16,17,18,19] and deep learning features [28]. Hand-designed features are commonly used for the color and texture features [15], SIFT features [16], and color names features [17], etc. At the same time, there are good representation capabilities in hand-designed features such as GOG [18] and LOMO [19]. In order to learn a robust distance measure, many scholars have proposed effective metric models, including KISSME [20], XQDA [19], FDA [21], etc. To fully understand the relevant algorithms to our proposed architecture in this paper, we will mainly introduce the research development of video-based person Re-ID and the current status of end-to-end deep learning algorithms in person Re-ID.

### 2.1. Video-Based Person Re-Identification

The research in video-based person re-identification is based on person Re-ID in multi-frame images. At present, more and more video-based person Re-ID methods are emerging. We believe that video-based person Re-ID can be divided into traditional methods and deep learning methods. In terms of traditional algorithms, the work of a past literature [2] uses the discriminative selection and ranking (DVR) method to select discriminative video fragments and extract their HOG3D features for matching. Another previous paper [3] proposes the STFV3D algorithm to extract spatiotemporal features (learn Fisher vectors) with spatial alignment. The top-push distance metric method [4] establishes a top-push constraint metric to improve the intra-distance and inter-distance between persons. In terms of deep learning algorithms, in a past paper [5], a novel recurrent neural network architecture is proposed to obtain space-time features in video. A previous literature [9] proposes an end-to-end learning architecture integrated by Convolutional Neural Networks (CNNs) and Bidirectional Recurrent Neural Networks (BRNNs) to match person in the video. In another past paper [22], a novel joint Spatial and Temporal Attention Pooling Network (ASTPN) is proposed as feature extractor to obtain features for video-based person Re-ID.

### 2.2. End-To-End Deep Learning on Person Re-Identification

With the wide applications of deep learning, end-to-end deep learning algorithms have appeared in many researches of person re-identification. The essence of an end-to-end learning algorithm is to completely connect the feature representation with the distance metric and jointly identify the same person. The binary input and the ternary input are the common strategy in person Re-ID algorithms of end-to-end learning. Literature [24] proposes a novel Deep Metric Learning (DML) method that jointly learns color features, texture features, and metrics in a unified framework. In a past paper [11], Chen et al. propose a novel deep end-to-end network to automatically learn the spatial-temporal fusion features, and utilize the Siamese to train sample pair. A previous work [25] presents a novel multi-channel parts-based Convolutional Neural Network (CNN) model under the triplet framework for person Re-ID. A different past work [26] also proposes a new end-to-end Comparative Attention Network (CAN) with triplet loss to learn the discriminative features of person images. For quaternary input, a past work [27] designs a quadruplet loss to ensure that model outputs have a larger interclass variation and a smaller intra class variation compared to the triplet loss. In our paper, we borrow the idea from the Siamese network of binary input, and employ two Siamese networks to learn the independent distance metric of different features. This effectively improves the performance of video-based person Re-ID.

## 3. The Proposed Hybrid End-To-End Deep Learning Architecture

### 3.1. Architecture Overview

**Figure 2:**
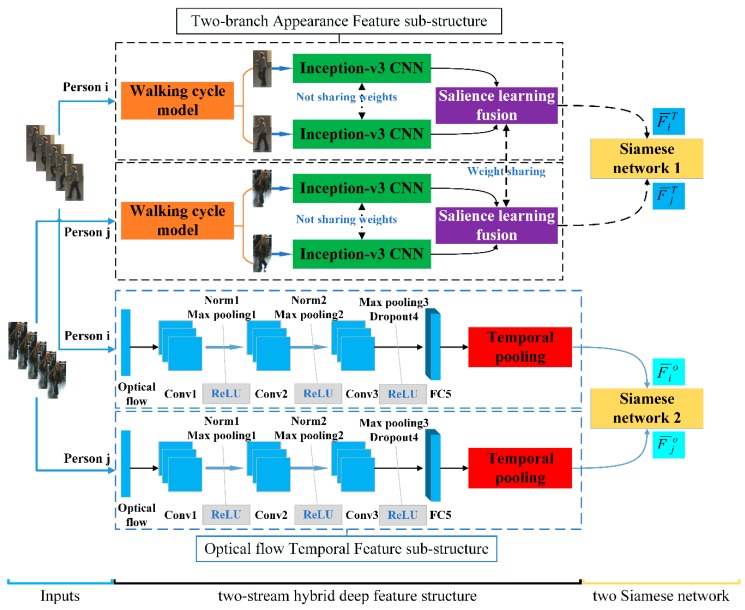
The framework of the proposed hybrid end-to-end deep learning architecture. The architecture consists of inputs, a two-stream hybrid deep feature structure, and two Siamese networks. The two-stream hybrid deep feature structure is composed of the Two-branch Appearance Feature sub-structure and the Optical flow Temporal Feature sub-structure, which can obtain abundant appearance information and stability temporal information of the pairwise person. Then, two Siamese networks are employed to compare the similarities between different persons of each type of feature.

The hybrid end-to-end deep learning architecture of our proposed method is shown in Figure 2. The hybrid end-to-end architecture consists of two-stream deep feature structure and two Siamese networks. The two-stream deep feature structure is composed of the Two-branch Appearance Feature sub-structure and the Optical flow Temporal Feature sub-structure, which can obtain abundant appearance information and stability temporal information of the pairwise person. It then employs two Siamese networks to compare the similarities between different persons of each type of feature.

In detail, for the first-stream feature substructure, we take the video of the original RGB image frames with persons i and j as inputs to the Two-branch Appearance Feature (TAF) sub-structure, respectively. A key process of the TAF sub-structure is that the walking cycle analysis model is used to select the pivotal frames N (N represents the number of pivotal frames) in the image sequence, then the pivotal frames are fed into a two-branch Incpetion-V3 network to learn the global appearance feature information. In addition, the fusion layer with weights sharing is applied to learn the salient and local features, whilst also fuse the global and local appearance information in the pivotal frames. Similarly, for the second-stream feature sub-structure, we use the optical flow image of video with persons i and j as inputs to the Optical flow Temporal Feature (OTF) sub-structure, respectively. Then, the CNN architecture and the temporal pooling generate temporal information. Finally, the obtained appearance features (${\bar{F}}_{i}^{T}$, ${\bar{F}}_{j}^{T}$) and temporal features (${\bar{F}}_{i}^{o}$, ${\bar{F}}_{j}^{o}$) are separately trained for similarity between features through two Siamese networks.

### 3.2. Input Data Acquisition

**Figure 3:**
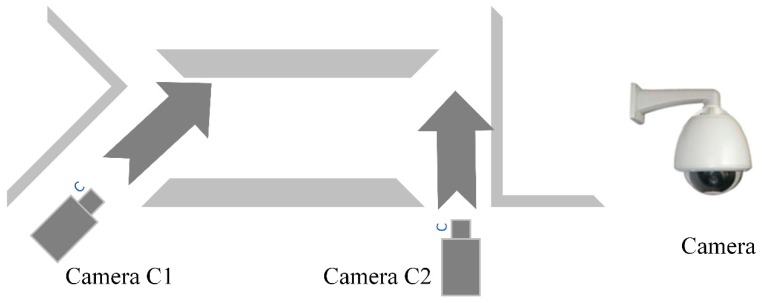
The data acquisition of multi camera visual sensor networks. These scenes are captured under disjoint cameras.

Multi-camera visual sensor networks are an important source of data acquisition for video-based person re-identification. The three public video datasets used in this paper all capture persons through multiple non-overlapping visual sensing cameras, as shown in Figure 3. Specifically, the PRID-2011 dataset [12] consists of image sequences extracted from multiple person trajectories recorded from two different static surveillance cameras. The iLIDS-VID dataset [13] is created from the persons observed in two non-overlapping camera views from the i-LIDS Multiple-Camera Tracking Scenario (MCTS) dataset which was captured at an airport arrival hall with a multi-camera CCTV network. The MARS dataset [14] was collected from six near-synchronized cameras on the campus of Tsinghua university. There were five 1080 × 1920 HD cameras and one 640 × 480 HD camera.

### 3.3. Two-Branch Appearance Feature Substructure (The First-Stream)

In order to select the pivotal frames in the videos and obtain more distinguishing global and local appearance features of persons, we designed a Two-branch Appearance Feature (TAF) substructure consisting of a walking cycle analysis model, a two-branch Inception-V3 network and a salience-learning fusion layer. The appearance feature sub-structure will be described in detail below.

#### 3.3.1. Walking Cycle Analysis Model

Given a video of the target person, in order to select reliable pivotal frames in the video and discard the interference frames, we consider employing the walking cycle analysis model [2,3,29] to obtain the pivotal frames as the input of the two-branch Inception-V3 network. This is done to prevent the appearance features are not affected by low-resolution image, complex background interference, and occlusion. In this model, we first extract the Flow Energy Profile (FEP) signal [2]. The FEP is a one-dimensional signal which represents the motion energy intensity induced by the activity of human muscles during walking [30], and is approximately estimated by optic flow computation. For each successive and raw RGB image frame, $r_{it}$ of person i in the video $S_{i}=\{r_{i1},r_{i2},\cdots ,r_{it},\cdots ,r_{iL}\}$, we calculate its flow energy by the connection between optical flow fields in the horizontal direction, $v_{x}$, and the vertical direction, $v_{y}$, as shown in Equation (1):

(1) $$E_{r_{it}}=\sum_{(x,y)\in p}{\left\|\left[v_{x}(x,y),v_{y}(x,y)\right]\right\|}_{2},$$

where $E_{r_{it}}$ represents the FEP value of the $r_{it}$-th frame, and p is an image of the lower body of a person.

**Figure 4:**
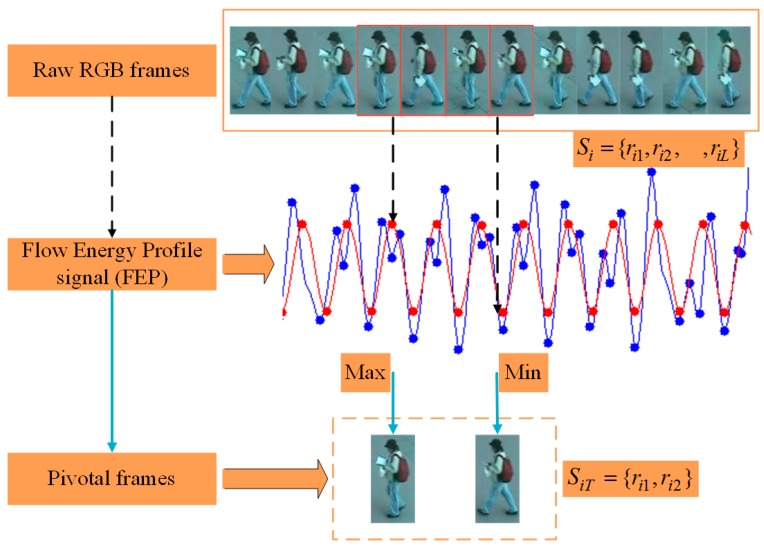
Pivotal frames extraction. The image frames with the golden box in the raw RGB frames is a partially selected video segment. The red curve in the Flow Energy Profile (FEP) signal is the regular FEP value and the blue curve is the rough FEP value.

The rough estimated FEP value of the walking cycle is prone to instability due to background noise and occlusion interference. According to this situation, the literature [3] uses the discrete Fourier transform method to convert the original FEP value into the frequency domain, thus obtaining a more accurate walking cycle model. In order to obtain a discriminative pivotal frame, we use this method [3] to convert the video sequence into a walking cycle. During the walking cycle, the image frames corresponding to the maximum and minimum FEP values can improve the result of person representation, so our paper selects them as the pivotal frames N (N represents the number of pivotal frames). As shown in Figure 4, from the graph of FEP value, the local maximum of energy value $E_{r_{it}}$ corresponds to the walking posture when the person’s legs overlap. Conversely, the local minimum value represents the person’s posture when their legs are farthest away. Through the analysis of the walking cycle model, the pivotal frames $S_{iPF}=\{r_{i1},r_{i2}\}$ are extracted from the video $S_{i}=\{r_{i1},r_{i2},\cdots ,r_{it},\cdots ,r_{iL}\}$ as the input of the two-branch Inception-V3 network.

**Remarks**. For the selection of pivotal frame’s number, our paper adopts the strategy of selecting even frames. Because the strategy of selecting the pivotal frames in our paper is to select the image frames corresponding to the maximum and minimum FEP values in the video, selecting the even pivotal frames not only discards the interference frames, but also preserves the complete appearance posture of the person.

#### 3.3.2. Two-BranchInception-V3 Network

Although the CNN has successfully demonstrated breakthroughs in person re-identification, changing the structure of the CNN from different perspectives enable achieve different performance. A straightforward way to improve CNN performance is to increase the number of layers in the network. Due to the deepening of the number of network layers, the number of network parameters and the computational cost will increase dramatically. At the same time, a deepened network and limited training samples may also cause serious overfitting problems. Hence, we construct the two-branch global feature learning module using the 42-layer Inception-V3 network [31]. Compared to other frameworks, such as VGG-Net [32] or Res-Net [33], we decided that the Inception-V3 network was more suitable for learning global features due to its high computational cost efficiency (higher modeling capacity at a smaller parameter size) and its capability for learning more discriminative appearance features at varying pivotal frames.

For the two-branch Inception-V3 network, although the two branches of model that learns the global features are the same Inception-V3 network, they do not share the weight parameters of the network. Intuitively, the pivotal frames $S_{iPF}=\{r_{i1},r_{i2}\}$ of person i are input into the two-branch Inception-V3 network, respectively, and the person’s global appearance features ($f_{i1}^{T}{}^{\prime }$ and $f_{i2}^{T}{}^{\prime }$) are obtained by learning.

**Remarks**. Since the selected pivotal frames correspond to different postures of the person walking in the video, and the interference frames with the occlusion are discarded, it is thus stable and easy to extract the person’s feature information from the pivotal frames with clear appearance. Furthermore, the two-branch Inception-V3 network can learn person’s appearance information in two different poses. The above observations are the main reasons for learning more discriminative appearance features at varying pivotal frames.

#### 3.3.3. Salience-Learning Fusion Layer

This fusion layer is designed to learn the local features and fuse the output of two-branch Inception-V3 network. Due to the phenomenon of feature redundancy in the feature map learned from the above multi-layer Inception-V3 network, and because some feature channels may capture interfering information about aperson, we suggest that the salience-learning fusion strategy can automatically discover and emphasize important local information, such as the head, torso, package, etc.

**Figure 5:**
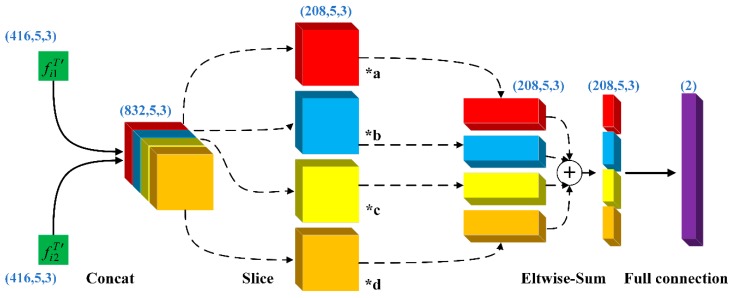
The process and detailed parameters of the salience-learning fusion layer. The blue font in the figure is the size of each layer input. The letters (“a” “b”, “c”, and “d”) are the weights learned.

As shown in Figure 5, we input features $f_{i1}^{T}{}^{\prime }$ and $f_{i2}^{T}{}^{\prime }$ into the salience-learning fusion layer, which can learn the different weights of each different feature channel. Then, we use the Eltwise operation to sum the feature channels. Finally, the information of each feature map is fused together through the fully connected layer, and we get the final appearance feature ${\bar{F}}_{i}^{T}$. This is the main reason why the layer can extract local appearance information and also reduce the feature dimension.

**Remarks**. In this work, we use the salience-learning fusion layer to exploit visual saliency. The strategy of salience-learning [26] has been successfully applied to person re-identification. As can be seen from Figure 8, the target person with the package can be accurately identified. Specifically, the layer combines the global and local features of a person in different poses, and the different weights of each feature channel can automatically calculate the positions in different visual saliency features. At the same time, the Eltwise-sum operation links the locations of local visual features.

### 3.4. Optical Flow Temporal Feature Substructure (The Second-Stream)

Although spatial appearance features are more discriminative than temporal features for person Re-ID [19], the temporal feature information can compensate for the errors caused by persons of similar appearance. The Optical Flow Temporal Feature (OTF) sub-structure combines the optical flow images with the CNN to obtain temporal features. The reason why the RNN is not added because the optical flow images contain temporal information associated with the pedestrian motion, and the temporal information learned in the optical flow images is mapped to the temporal feature map, no longer use the RNN to get the temporal information. Finally, the temporal pooling method is used to aggregate the sequence-level temporal features into a single temporal feature. The OTF sub-structure is described in detail later in this section.

#### 3.4.1. The CNN Architecture

In this paper, the input of the OTF sub-structure (the second-stream) is the image frame of the optical flow information corresponding to the video, which is the same as the literature [11]. Specifically, we define the optical flow images in the video $S_{io}$ as $S_{io}=\{o_{i1},o_{i2},\cdots ,o_{it},\cdots ,o_{iL}\}$, where $o_{iL}$ represents the optical flow image and L is the video length. The method of obtaining $o_{it}$ is the same as the method described in Section 3.3, computed using the Lucas–Kanade optical flow technique [34].

As shown in Figure 2, we employed a previously proposed CNN architecture [11] to obtain the temporal information. However, in our case, some of the parameters in the architecture were modified. Figure 2 shows the network architecture, and Table 1 demonstrates the parameters of this CNN architecture. The CNN architecture is composed of three convolutional layers, two fully connected layers and a dropout layer. Note that the process steps of each convolutional layer are convolutional, nonlinear activation functions, and pooling. We chose the rectified linear unit (ReLU) as the activation function and set the pooling operation to max-pooling. We input the optical flow image frames $S_{io}=\{o_{i1},o_{i2},\cdots ,o_{it},\cdots ,o_{iL}\}$ of person i into the CNN architecture and generate the output temporal feature vector $F_{i}^{o}=\{f_{i1}^{o},f_{i2}^{o},\cdots ,f_{it}^{o},\cdots ,f_{iL}^{o}\}$ after passing the CNN. The process of the above CNN architecture can be expressed by Equations (2) and (3) as follows

(2) $$f_{it}^{o}{}^{\prime }=Maxpool(relu(Conv(o_{it}))),1\leq t\leq L,$$

(3) $$f_{it}^{o}=connect(f_{it}^{o}{}^{\prime }),1\leq t\leq L,$$

where $o_{it}$ denotes the optical flow image at t moment, $f_{it}^{o}{}^{\prime }$ is the feature vector through three convolutional layers, and $f_{it}^{o}$ is the temporal feature vector after the CNN architecture.

#### 3.4.2. Temporal Pooling

To aggregate the temporal information of all time steps in the OTF sub-structure, the multi-frame feature vector is aggregated into a single feature vector using the temporal pooling method. The implementation of these functions can be achieved by mean pooling, max pooling, and sum pooling, but it was proven [5] that mean pooling is more suitable for aggregating information in person Re-ID. The median value is tested as well to remove gross errors. In our paper, we adopt the same temporal mean-pooling method to take the temporal feature vector $F_{i}^{o}=\{f_{i1}^{o},f_{i2}^{o},\cdots ,f_{it}^{o},\cdots ,f_{iL}^{o}\}$ from the CNN architecture as inputs, and then produce a single feature vector ${\bar{F}}_{i}^{o}$ to represent the final temporal feature of person i in the video. This process can be expressed by the following Equation (4).

(4) $${\bar{F}}_{i}^{o}=\frac{1}{L}\sum_{t=1}^{L}f_{it}^{o},$$

where $f_{it}^{o}$ is the temporal feature at time t, and t∈[1,L]. ${\bar{F}}_{i}^{o}$ is the final temporal feature of person i generated by the OTF sub-structure.

### 3.5. Two Siamese Networks

The Siamese network is a measure of the similarity of two objects, which consist of two substructures with shared weights [35]. Each substructure is used as a feature extractor to output the trained feature vectors, and the Siamese network compares these feature vectors using Euclidean distance. The essence of this network comparison idea is to try to reduce the distance between feature vectors of the same class and increase the distance between feature vectors of different classes. Thus, the similarity of a pair of inputs is distinguished by a margin. Fortunately, this property is close to the distance metric learning algorithm in the person Re-ID, so the Siamese network has been applied to person Re-ID work. For video-based person Re-ID, the Siamese network can use the features of a pair of image sequences to train similarity.

Concretely, in our paper, as shown in Figure 2, we constructed two Siamese network [8] to learn the independent distance of the TAF sub-structure and OTF sub-structure, respectively. For the first Siamese network, the final appearance feature vector ${\bar{F}}_{i}^{T}$ and ${\bar{F}}_{j}^{T}$ obtained by the pivotal frames of person i and j through the TAF substructure are taken as inputs. The similarity loss function Sim(•) of the generic first Siamese network is defined as follows

(5) $$Sim({\bar{F}}_{i}^{T},{\bar{F}}_{j}^{T})=\left\{ \begin{array}{l} \frac{1}{2}{\left\|{\bar{F}}_{i}^{T}-{\bar{F}}_{j}^{T}\right\|}^{2}\;i=j\\ \frac{1}{2}[\max(M-\left\|{\bar{F}}_{i}^{T}-{\bar{F}}_{j}^{T}\right\|,0){]}^{2}\;i\neq j \end{array}\right.,$$

where M represents the margin value in the Siamese network. Similarly, the second-stream Siamese pseudo-network employs the same function with different types of feature vector inputs, as shown in Equation (6):

(6) $$Sim({\bar{F}}_{i}^{o},{\bar{F}}_{j}^{o})=\left\{ \begin{array}{l} \frac{1}{2}{\left\|{\bar{F}}_{i}^{o}-{\bar{F}}_{j}^{o}\right\|}^{2}\;i=j\\ \frac{1}{2}[\max(M-\left\|{\bar{F}}_{i}^{o}-{\bar{F}}_{j}^{o}\right\|,0){]}^{2}\;i\neq j \end{array}\right.,$$

It should be noted that ${\bar{F}}_{i}^{o}$ and ${\bar{F}}_{j}^{o}$ denote the temporal feature vectors of person i and j. To sum up, the joint objective function $Sim_{obj}$ combined with the two-stream Siamese network is shown in Equation (7):

(7) $$Sim_{obj}=\partial_{T}Sim({\bar{F}}_{i}^{T},{\bar{F}}_{j}^{T})+\partial_{o}Sim({\bar{F}}_{i}^{o},{\bar{F}}_{j}^{o}),$$

where $\partial_{T}$, $\partial_{o}$ represents the loss weight, and $\partial_{T}>\partial_{o}$. The reason for setting these weights is the effectiveness of the appearance features compared to the temporal features.

## 4. Training and Test

### 4.1. Training (Joint Multiple Loss)

**Figure 6:**
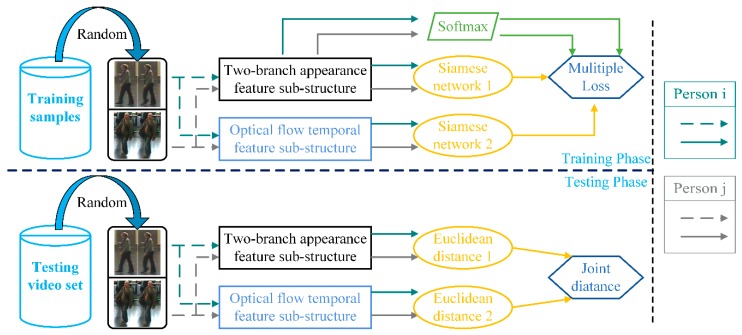
The training steps and test steps of the proposed hybrid end-to-end deep learning architecture. The upper left side of the figure is the training phase, and the lower-left side is the test phase. The right side of the figure is an annotation of the arrows of different colors.

During the training phase, we adopted a joint training method similar to those previously described in the literature [11]. The core of this strategy is to combine the objective function of two Siamese networks and the objective function of the predicted person’s identity to train our proposed appearance feature learning substructure. In order to take full advantage of label information, we used the Softmax loss function [36] to predict the person’s identify. In our work, as shown in the Figure 6 for feature vector ${\bar{F}}_{i}^{T}$ in the first-stream substructure, the posterior probability of predicting person i is as follows

(8) $$P_{iT}({\tilde{m}}_{i}=m_{i}|S_{i})=\frac{\exp(W_{m_{i}}{\bar{F}}_{i}^{T})}{\sum_{n=1}^{n_{id}}\exp(W_{n}{\bar{F}}_{i}^{T})},$$

where $m_{i}$ represents the category label of the person i training video sample $S_{i}$, ${\tilde{m}}_{i}$ is the predicted label, and $W_{n}$ refers to the Softmax function parameter of the person’s class n. The training loss is computed as

(9) $$Loss_{i}^{soft}=\sum \log(P_{iT}({\tilde{m}}_{i}=m_{i}|S_{i})),$$

Note that the use of the Softmax loss function during training is only for the appearance feature sub-structure (the first-stream).

Therefore, the loss function Loss of the entire architecture is as follows

(10) $$Loss=Sim_{obj}+Loss_{i}^{soft}+Loss_{j}^{soft},$$

where $Loss_{i}^{soft}$ and $Loss_{j}^{soft}$ are the Softmax functions of persons i and j, respectively.

### 4.2. Test (Re-Identification)

In the testing phase, given a probe video and candidate video set, the test of the pedestrian re-identification algorithm is compared the distance between the probe video and each the videos in the candidate video set. Therefore, our goal is to calculate and rank the distances between the person’s features. In our work, as shown in Figure 6, we use Euclidean distance to express the similarity of persons. For the final appearance feature vectors (${\bar{F}}_{i}^{T}$ and ${\bar{F}}_{j}^{T}$) and temporal feature vectors (${\bar{F}}_{i}^{o}$ and ${\bar{F}}_{j}^{o}$), the independent Euclidean distances are calculated as follows

(11) $$d^{T}=\left\|{\bar{F}}_{i}^{T}-{\bar{F}}_{j}^{T}\right\|,$$

(12) $$d^{o}=\left\|{\bar{F}}_{i}^{o}-{\bar{F}}_{j}^{o}\right\|,$$

where $d^{T}$ and $d^{o}$ represent the distance of appearance features and the distance of temporal features, respectively. Finally, the weighting merges the above distances and sorts them:

(13) $$d=\partial_{T}d^{T}+\partial_{o}d^{o},$$

where d is the joint distance between persons. The data selection principle for training phase and testing phase is specified in Section 5.1.3.

## 5. Experiments

In this section, we evaluated the proposed architecture of the three video datasets. The first part of our experimental work was mainly to compare experiments with other algorithms, and the other part was to verify the effectiveness of some factors in the proposed method.

### 5.1. ExperimentalSetup

#### 5.1.1. Datasets

**Figure 7:**
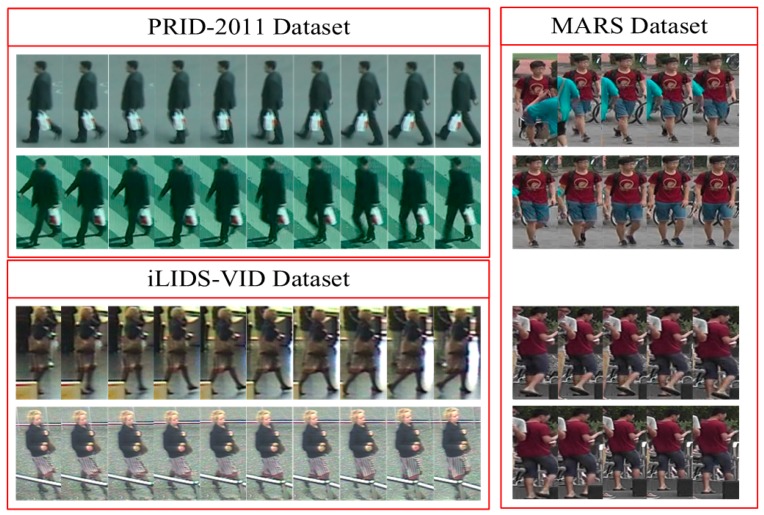
Some samples of persons from different camera-views in three public datasets, including the PRID-2011 dataset, the iLIDS-VID dataset and the Motion Analysis and Re-identification Set(MARS) dataset.

The details of the three datasets are as follows, and Table 2 and Figure 7 show the basic information and some person samples, respectively.

The PRID-2011 dataset [12] is composed of images captured by two cameras (A and B) from outdoor non-overlapping perspectives. There are 385 identities and 749 identities in cameras A and B, respectively, and 200 persons with the same identity under both cameras. Note that there are 400 video sequences for 200 subjects. The video sequence length of each pedestrian is between 5 and 675 frames. The design peculiarity of this dataset is the challenges of persons with simple background interference, less occlusions, and lighting variations.

The iLIDS-VID dataset [13] consists of 600 video sequences of 300 identities, also captured from two non-overlapping cameras view. Each video sequence of the dataset is between 23 and 192 frames in length. The challenges of this dataset include camera-view changes, illumination variations, complex cluttered background, and serious occlusions.

The MARS dataset [14] is a relatively new video person Re-ID dataset. The dataset is derived from an extension of the Market1501 dataset [37] with 1261 pedestrians and 20,478 tracklets. These tracklets were captured by six cameras and collected using a DPM detector [38] and a GMMCP tracker [39]. Furthermore, there are 3278 distracted tracklets in the dataset due to false detection and association.

#### 5.1.2. Evaluation Protocol

In order to evaluate the effectiveness of the person Re-ID algorithm, we adopted the cumulative matching characteristic (CMC) curve [40] and the mean average precision (mAP) [14] as evaluation criteria. The CMC value refers to the expectation of a correct match in the rank-k (%) position. The CMC curve refers to the curve of correct match results in the rank-k (%). The mAP considers both the precision and recall of multiple same persons in a gallery. For the PRID-2011 dataset and iLIDS-VID dataset, we used the CMC value to evaluate the performance of the algorithm. For the MARS dataset, both CMC curve and mAP were adopted. The experimental results were the average values after ten random experiments.

#### 5.1.3. Implementation Details

In terms of data preparation, we followed an experimental data selection principle similar to the literature [13] on the PRID-2011 dataset and the iLIDS-VID dataset. Specifically, we used the 177 persons out of 354 videos from the camera A and B on the PRID-2011 dataset. On the iLIDS-VID dataset, we used the 400 videos of 200 persons for the experiment. Similarly, we randomly selected the videos from one camera-view for the training samples, and other videos from the other camera-view for testing. Finally, for the MARS dataset, we followed the experimental data selection principle as described in the literature [14]. The dataset was divided into 625 persons for training, and the rest of the persons for testing. In addition, since we consider the pairwise input of the Siamese network, the person’s videos in the training set were randomly combined into positive sample pairs and negative sample pairs. The sequence length on the three datasets was set to 16, as in the literature [11]. In cases where the person sequence was shorter than 16, we use the entire sequence.

In terms of architecture parameter settings, our experiments were conducted under the Caffe [41] deep learning framework. When we trained the deployed network architecture on the deep learning framework, some necessary training parameters needed to be set: initial learning rate was set to 0.0001, the momentum to 0.9, max iterations to 30,000, and the learning rate decline policy was “inv”. Then, the M (margin value of the Siamese network) was set to 2. Lastly, the optimization method during training was the stochastic gradient descent method.

### 5.2. Comparative Experiment

In order to verify the performance of the proposed architecture on the PRID-2011 dataset, iLIDS-VID dataset, and MARS dataset, we established a comparative experiment to compare our video-based person Re-ID architecture with other state-of-the-art algorithms.

#### 5.2.1. Results on PRID-2011 Dataset

**Figure 8:**
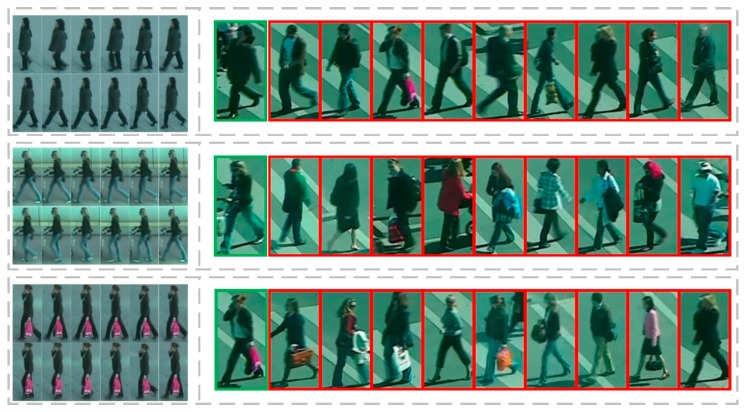
The re-identification results of some people in the proposed architecture in the PRID-2011 dataset. The first column in the figure represents the probe video. The second column is the result of sorting the top ten with the distances, where the green boxes indicate the first and the same person, and the red boxes are the wrong match.

For the PRID-2011dataset, we compared the performance of our proposed architecture with eleven state-of-the-art methods, including DVR [2], DVDL [42], STFV3D [3], RMLLC-SLF [43], TDL [4], RFA [44], CNN-RNN [5], CNN-BRNN [9], CRF [10], ASTPN [22], TSSCN [11], and TAM-SRM [45]. The experimental results in the CMC values are shown in Table 3. The black bold in Table 3 indicates the highest correct recognition rate. Note that among these approaches, the first four methods are based on the traditional person Re-ID method, and the remaining are based on deep learning algorithms. As can be seen from Table 3, Rank-1, Rank-5, and Rank-20 of our proposed method reached 79%, 92%, and 99%, respectively. In the comparison methods, in addition to the TAM-SRM algorithm, the Rank-1 recognition rate was improved compared to the rest of the algorithms. Concurrently, our method was also 1% higher on Rank-1 than the similar TSSCN method. These results all show the good performance of our proposed algorithm on the PRID-2011 dataset. Remarks, Figure 8 shows the re-identification sorting results of some persons in the PRID-2011 dataset.

#### 5.2.2. Results on iLIDS-VID Dataset

For the iLIDS-VID dataset, the comparison method we used was consistent with the experiment on the PRID-2011 dataset. The experimental results are shown in Table 4, the black bold indicates the best recognition rate. Rank-1, Rank-5, and Rank-20 of our method on the iLIDS-VID dataset reached 59%, 82%, and 96%, respectively. Compared to the comparison method, our method had a slight gap with the CRF method, the TSSCN method, and the ASTPN method. Our analysis considered that the number of training samples in the dataset was small, and the challenges were complex, including background interference and severe occlusion.

#### 5.2.3. Results on MARS Dataset

**Figure 9:**
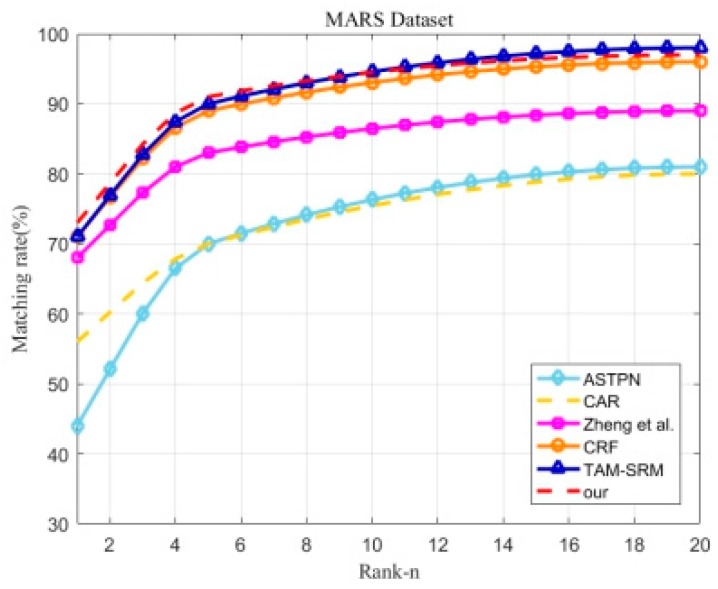
Experimental results of the comparison with other state-of-the-art algorithms for the MARS dataset in term of the CMC curve. Because the work of Li et al. [46] only reported the Rank-1 value, their results cannot be drawn as a curve.

The MARS dataset is a large-scale dataset for video-based person Re-ID; we compared our method with six state-of-the-art methods, including the ASIPN [22], CAR [29], Zheng et al. [14], CRF [10], TAM-SRM [45], and Li et al. [46]. The experimental results are shown in Table 5 and Figure 9, and the bold black indicates the highest recognition rate. Rank-1, Rank-5, and Rank-20 of our method for the MARS dataset reached 73%, 91%, and 97%, respectively. In the comparative method, the framework proposed in this paper outperformed the TAM-SRM method by 2% and 1% on Rank-1 and Rank-5, respectively. Simultaneously, our method was also superior to the method of Zheng et al. [14] and the TAM-SRM algorithm in terms of the mAP evaluation criterion. The above results indicate that our architecture had good performance on the MARS dataset. In particular, the method of Li et al. [46] obviously outperformed the proposed algorithm by a large margin. The method of Li et al. [46] takes full advantage of the labeled information of the pretrained model on image-based person re-identification datasets to train. The results provide evidence that we can improve the training ability of video-based person re-identification models by using labeled information on image-based datasets.

### 5.3. Verification Experiment ofKey Components

In this section, we performed in-depth experiments on the PRID-2011 dataset to verify the effectiveness of four key components, including the pivotal frame’s number, the different Inception-V3 structure and network, the different weights with two-stream architecture, and the independent effectiveness of each stream feature’s sub-structure. The specific experimental results and analysis are as follows. Note that, when we verified the effectiveness of one component, the other two components were kept unchanged. Therefore, we changed this component to conduct the verification experiment.

#### 5.3.1. Effectiveness of the Pivotal Frame’s Number

As shown in the experimental results of the first to third rows in Table 6, the selection of different numbers of pivotal frames yielded different recognition rates. We can observe that when the number of pivotal frames equals 2, N=2, the best performance and recognition rates were achieved. Note that we ensured that the other two components were “Our (Inception-V3-5c)” and “Our ($\partial_{T}=0.7,\partial_{o}=0.3$)” when we completed the experiment. The experimental results also show that the number of pivotal frames is an important factor in the appearance feature substructure of our proposed. Simultaneously, pivotal frames also help the TAF model get better appearance feature. For the effectiveness of the pivotal frame’s number, considering the complete appearance feature sub-structure, the increase in the number of pivotal frames is only the repeated accumulation of the two walking postures of a person. Minor changes in the appearance of the person are likely to cause the fitting of the global feature representation on the deep Inception-V3 network, and the increase in the number of pivotal frames may increase the likelihood of similar poses between different persons.

#### 5.3.2. Effectiveness of the Different Inception-V3 Structures and Different Network

In order to verify that the Inception-V3 network can extract distinguishing features for pivotal frames, we compared different Inception-V3 structures with the Res-Net (50) network [33]. Comparing the results of lines 1–4 in Table 7 we can see that using the Inception-V3 network to perform the extraction of appearance features consistently improves the matching performance. Note that “Inception-V3-3c”, “Inception-V3-4e”, and “Inception-V3-5c” refer to the outputs of the “3c”, “4e”, and “5c” modules in the Inception-V3 network, respectively. In particular, the "Inception-V3-5c" structure in the Inception-V3 network performed better than the rest of structure, with improvements of approximately 14% and 8% on Rank-1, respectively. These results verify that the “Inception-V3-5c” structure can learn a rich global appearance feature and effectively improve the person Re-ID recognition rate.

#### 5.3.3. Effectiveness of the Different Weights with Two-Stream Architecture

In the hybrid end-to-end learning architecture, the appearance features and temporal features of persons can be extracted separately. In order to verify the importance of each stream feature structure, from the 1 to 5 rows in Table 8, we performed a verification experiment of two streams networks with five different weights. It can be seen that when the weight is $\partial_{T}=0.7,\partial_{o}=0.3$, the optimal result of our architecture was 79% for Rank-1. Note that when there was no temporal feature (OTF) sub-structure, the Rank-1 recognition rate was 70%. After adding the temporal feature (OTF) sub-structure, the recognition rate was significantly improved. The experimental results prove that the temporal features of the OTF model are beneficial to the method proposed in video-based person Re-ID.

#### 5.3.4. Independent Effectiveness of Each Stream Feature Substructure

In this subsection, we performed a comparison experiment on the PRID-2011 dataset to verify the independent effectiveness of each stream feature’s sub-structure. From rows 1 to 5 in Table 9, we chose the independent feature substructure (TAF sub-structure and OTF sub-structure) to be compared with related algorithms, including CNN-RNN [5], CNN-BRNN [9], and CRF [10]. The results showed that the TAF sub-structure reaches 70%, 88%, and 95% on Rank-1, Rank-5, and Rank-20, respectively. Compared with the three other algorithms, the results of the independent TAF sub-structure were better than the CNN-RNN algorithm, and lower than the other two algorithms. For the independent OTF sub-structure, the Rank-1, Rank-5, and Rank-20 reached 57%, 74%, and 89%, respectively. However, the results of the OTF structure were lower than results of the other three algorithms. Among the three algorithms, they all use appearance feature information and temporal feature information to represent the person.

## 6. Conclusions

In this paper, we proposed a hybrid end-to-end deep learning architecture for video-based person re-identification. The architecture consists of the two-stream hybrid feature structure and two Siamese networks. The two-stream hybrid deep feature structure includes the Two-branch Appearance Feature sub-structure and the Optical flow Temporal Feature sub-structure, which can separately learn appearance and temporal information. For the video-based person re-identification, our method showed, in a large number of experiments on three datasets, that separate feature structures were superior in their ability to learn appearance features and temporal features, as well as the independent distances of different modal features. In future, we will add semantic features to enrich the feature learning model and improve the loss function to optimize the distance metric.

## Figures and Tables

**Table 1:** Parameters of the CNN architecture.

Layers	Network Parameter/Type
Conv1	Filter 5 × 5/stride 2/pad 4
Max pool1	Filter 2 × 2/stride 2
Conv2	Filter 5 × 5/stride 2/pad 4
Max pool2	Filter 2 × 2/stride 2
Conv3	Filter 5 × 5/stride 2/pad 4
Max pool3	Filter 2 × 2/stride 2
Dropout	dropout ratio 0.5

**Table 2:** Detailed information of the experimental datasets.

Dataset	Persons	Cameras	Videos	Resolution	Evaluation
PRID-2011	385/749	2	400	64 × 128	CMC
iLIDS-VID	300	2	600	64 × 128	CMC
MARS	1261	6	20,478	128 × 256	CMC & mAP

**Table 3:** Comparison experiment with two types of state-of-the-art algorithms for the PRID-2011 dataset in terms of CMC values.

PRID-2011 Dataset				
Category	Methods	Rank-1	Rank-5	Rank-20
Traditional	DVR	40	72	92
	DVDL	40	70	86
	STFV3D	42	72	92
	RMLLC-SLF	50	78	97
	TDL	57	80	94
Deep Learning	RFA	58	86	98
	CNN-RNN	65	90	97
	CNN-BRNN	72	92	98
	CRF	77	93	98
	ASTPN	77	**95**	99
	TSSCN	78	94	99
	TAM-SRM	79	94	99
	Our	**79**	92	**99**

**Table 4:** Comparison experiment with two types of state-of-the-art algorithms for theiLIDS-VID dataset in terms of CMC values.

iLIDS-VID Dataset				
Category	Methods	Rank-1	Rank-5	Rank-20
Traditional	DVR	40	61	82
	DVDL	26	48	69
	STFV3D	37	64	87
	RMLLC-SLF	59	85	96
	TDL	56	88	98
Deep Learning	RFA	49	76	90
	CNN-RNN	65	**90**	97
	CNN-BRNN	55	85	95
	CRF	61	85	97
	ASTPN	**62**	86	**98**
	TSSCN	60	86	97
	TAM-SRM	55	87	97
	Our	59	82	96

**Table 5:** Comparison experiment with other state-of-the-art algorithms on the MARS dataset in terms of CMC values and mean average precision (mAP).

MARS Dataset				
Methods	Rank-1	Rank-5	Rank-20	mAP
ASTPN	44	70	81	-
CAR	56	70	80	-
Zheng et al.	68	83	89	49.3
CRF	71	89	96	-
TAM-SRM	71	90	**98**	50.7
Li et al.	**82**	-	-	**65.8**
Our	73	**91**	97	52.4

**Table 6:** Verification experiment results for the pivotal frame’s number for the PRID-2011 dataset in terms of CMC values.

PRID-2011 Dataset			
Methods	Rank-1	Rank-5	Rank-20
Our (N=2)	**79**	**92**	**99**
Our (N=4)	72	90	96
Our (N=6)	73	85	92
Our (N=8)	73	89	93

**Table 7:** Verification experiment results for the different Inception-V3 structures and different networks on the PRID-2011 dataset in terms of CMC values.

PRID-2011 Dataset			
Methods	Rank-1	Rank-5	Rank-20
Res-Net (50)	66	82	90
Our(Inception-V3-3c)	65	85	95
Our(Inception-V3-4e)	71	88	95
Our(Inception-V3-5c)	**79**	**92**	**99**

**Table 8:** Verification experiment results for the different weights with the two-stream architecture on the PRID-2011 dataset in terms of CMC values.

PRID-2011 Dataset			
Methods	Rank-1	Rank-5	Rank-20
Our($\partial_{T}=0.5,\partial_{o}=0.5$)	73	88	95
Our($\partial_{T}=0.6,\partial_{o}=0.4$)	76	**93**	97
Our($\partial_{T}=0.7,\partial_{o}=0.3$)	**79**	92	**99**
Our($\partial_{T}=0.8,\partial_{o}=0.2$)	77	92	97
Our($\partial_{T}=1,\partial_{o}=0$)	70	86	93

**Table 9:** Verification experiment results for the independent effectiveness of each stream feature substructure on the PRID-2011 dataset in terms of CMC values.

PRID-2011 Dataset			
Methods	Rank-1	Rank-5	Rank-20
Our ($\partial_{T}=1,\partial_{o}=0$)	70	86	93
Our ($\partial_{T}=0,\partial_{o}=1$)	57	74	89
CNN-RNN	65	90	97
CNN-BRNN	72	92	98
CRF	**77**	**93**	**98**
